# A Novel Permutation Entropy-Based EEG Channel Selection for Improving Epileptic Seizure Prediction

**DOI:** 10.3390/s21237972

**Published:** 2021-11-29

**Authors:** Jee S. Ra, Tianning Li, Yan Li

**Affiliations:** School of Sciences, University of Southern Queensland, Toowoomba, QLD 4350, Australia; jee.ra@usq.edu.au (J.S.R.); Yan.Li@usq.edu.au (Y.L.)

**Keywords:** EEG channel selection, permutation entropy, K nearest neighbors (KNN), support vector machine (SVM), genetic algorithm (GA)

## Abstract

The key research aspects of detecting and predicting epileptic seizures using electroencephalography (EEG) signals are feature extraction and classification. This paper aims to develop a highly effective and accurate algorithm for seizure prediction. Efficient channel selection could be one of the solutions as it can decrease the computational loading significantly. In this research, we present a patient-specific optimization method for EEG channel selection based on permutation entropy (PE) values, employing K nearest neighbors (KNNs) combined with a genetic algorithm (GA) for epileptic seizure prediction. The classifier is the well-known support vector machine (SVM), and the CHB-MIT Scalp EEG Database is used in this research. The classification results from 22 patients using the channels selected to the patient show a high prediction rate (average 92.42%) compared to the SVM testing results with all channels (71.13%). On average, the accuracy, sensitivity, and specificity with selected channels are improved by 10.58%, 23.57%, and 5.56%, respectively. In addition, four patient cases validate over 90% accuracy, sensitivity, and specificity rates with just a few selected channels. The corresponding standard deviations are also smaller than those used by all channels, demonstrating that tailored channels are a robust way to optimize the seizure prediction.

## 1. Introduction

Epilepsy is a serious brain disorder, second only to strokes in its effect. More than 50 million people worldwide are affected by epilepsy, and the symptoms of one-third of those are not controlled by anticonvulsant medication. Therefore, one of the critical objectives in seizure management in epileptic patients is its early detection and prediction to provide well-timed preventive interventions [1]. If epileptic seizures can be predicted in advance, the patients’ unfortunate consequences can be alleviated. Unfortunately, despite decades of international efforts devoted to predicting seizures, seizure prediction remains an unsolved problem [2].

Two key components in research into seizure detection and prediction using epileptic electroencephalography (EEG) signals are feature extraction and classification [3,4]. Most of the existing research is patient-independent and trains models for all types of patients [5,6,7,8,9,10], while some EEG-based seizure detection algorithms are patient-dependent and are adaptive to individual patients. In order to reduce the computational load for a real-time seizure prediction using EEG data, identifying the most relevant channels for the seizure prediction is both important and effective. It can make seizure-predicting wearable or implantable devices with less complicated feature extraction during the process of developing machine learning algorithms for the real-time analysis. In addition, a decreased number of EEG channels may deliver more convenience to the patients.

However, selecting channels in epileptic features extraction is often not considered necessary. As to patient-specific feature extraction, although the benefits of patient-specific seizure prediction research have not yet been identified, we believe that discovering well-chosen channels tailored to an individual can lead to the uncovering of behavioral patterns in seizure activity through relations between neurophysiological characteristics and EEG channels [11], given the complex aspects of seizure onsets.

Even though much epileptic EEG feature-extraction research has been published, not many papers related to EEG channel selection have been reported over the last decades. Furthermore, the research about machine learning performance comparisons between results with selected channels and all channels is seldom found. Chang et al. [12] proposed that channel selection reduced the channel number from 22 to fewer than 6 channels, and it also saved 93.73% of the computation time. The best result showed a success rate of 70% in three-channel cases of the EEG database. Ibrahim et al. [13] also showed the seizure prediction probability by the selected channel, and the selected feature was higher than 70%, while the false-alarm probability was less than 30%. The channels were classified by a statistical frame. Chakrabarti et al. [14] applied an artificial neural network (ANN) and a principal component analysis (PCA) for the selection of epileptic EEG channels. The results revealed that the accuracy decreased simultaneously as the number of channels decreased. The highest accuracy of 86.7% was achieved with 18 channels out of 23 channels.

Nevertheless, none of those studies showed the machine learning validating performance comparisons between results with selected channels and results with all channels. Moctezuma and Molinas [15] decomposed the EEG data from each channel into different frequency bands using the empirical mode decomposition (EMD) or the discrete wavelet transform (DWT) for the channel selection. The results showed accuracies of up to 100% with only one EEG channel in the epileptic seizure classification, while all the test results of channels were less than 100%; however, this research only classified the seizure and non-seizure signals, not the pre-ictal signals. The classification performance to detect seizure EEG signals usually achieves high accuracy. Prasanna et al. [16] examined recent research to classify between seizure and non-seizure EEG signals. According to their review, the accuracy range that recent studies achieved was from 90% to almost 100%. This research, however, focuses on seizure prediction instead of seizure detection.

In this research, we confine the features to the channels, and present a patient-dependent optimization method for EEG channel selection based on the permutation entropy (PE) values, and employing K nearest neighbors (KNN) combined with a genetic algorithm (GA) for epileptic-seizure prediction. In the last few decades, some seizure prediction studies have applied the GA to generate solutions to search features derived from EEG signals [17,18,19,20,21,22]. For example, Firpi et al. [23] employed a GA to create artificial features from EEG signals. In their experiment, three patients’ datasets were used, and the validation was performed by the KNN, achieving an average of 83.33% seizure prediction. KNN is one of the most widespread methods in the machine learning techniques. As medical facilities require minimal computational time, the KNN has been used as a seizure prediction algorithm in many recent studies [24,25,26,27]. For instance, Wang et al. [27] proposed a KNN analysis on EEG data from 10 patients with epilepsy, achieving 73% sensitivity and 67% specificity on average using a 150-min prediction horizon.

The classifier in this research is the SVM, as the SVM classification complexity does not depend on the feature dimension, and it provides a global solution [28,29,30], which might be appropriate for epileptic EEG classification. Shiao et al. [31] showed that the SVM-based seizure prediction system could achieve a robust prediction for preictal period and normal period iEEG signals from dogs with epilepsy. The sensitivity was 90–100%, and the false-positive rate was about 0–0.3 times per day. However, SVM does not always seem suitable for the epileptic EEG signals classification. Direito et al. [32] used massive data from 216 patients from the European Epilepsy Database, including 185 patients with scalp EEG recordings and 31 with intracranial data. They tested their method over a total of 16,729.80 h with inter-ictal data, including 1206 seizures using the SVM. The method achieved an overall sensitivity of 38.47% and a false-positive rate per hour of 0.20 (statistical significance only in 11% of the patients). This disproved the importance of proper feature extractions.

This research is the first study to compare the effectiveness of EEG channel selection with that before channel selection. It also aims to reveal that patient-specific channel selection can contribute to a more efficient seizure prediction. The remainder of this paper is arranged as follows. Section 2 presents the details of the proposed techniques for the EEG channels selection and classifications. Section 3 explains the datasets used in this paper, experimental setup, and results. Section 4 discusses the findings of this research. Finally, the conclusions of this study are drawn in Section 5.

## 2. Methodology

**Figure 1:**
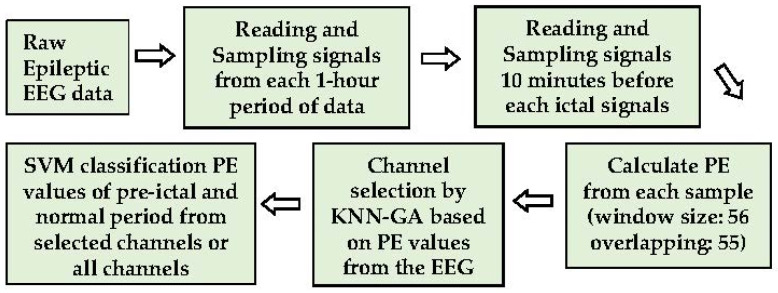
The main process of methods.

The goal is to construct a less complicated seizure prediction system with less computational load but high accuracy for real-time seizure prediction. The PE values differentiated by KNN combined with a GA (KNN-GA) are employed in this research to select channels for efficient analysis and seizure prediction. The overall process is divided into three steps: PE calculation and data sampling, channel selection by KNN-GA, and test modelling by the machine learning method, SVM. Firstly, the raw EEG signals without noise-filtration, segmented into time windows, are directly used to acquire the PE values, which are the parameters obtained by feature extraction. Secondly, the selected PE values of each channel are used for selecting the most pre-ictal related channels through KNN-GA, which is executed repeatedly (maximum number of executions is 30 in this study). Finally, the effect of the selected channels is validated and compared using the SVM classification with all 23 channels. The primary process of the method is illustrated below (Figure 1).

### 2.1. Permutation Entropy

For the proper channels to be selected efficiently from EEG signals in the dataset, the collected original data samples are used as the input to obtain the PE values to measure the detailed variations in the EEG signals by expressing the signal in multi-scale time-frequency domains. The PE provides a quantity measure of the complexity of a dynamic system by capturing the order relations on time-series signals and their probability distribution of the ordinal patterns [33].

The first step is to convert a one-dimensional time series into a matrix of overlapping column vectors. Then, M-dimensional vectors are mapped into unique permutations that achieve the ordinal rankings of the data. These permutations are the values that are associated with each partitioned vector based on the ordinal position of the values within the vector. Then, the relative frequency of each permutation is calculated by counting the number of times the permutation is found in the signals divided by the total number of sequences [34]. Finally, the relative frequency of each permutation is used to compute the PE of the order M of the signals, which is given by Equation (1) [34]:

(1) $$PE_{M}=-\overset{M!}{\underset{i=1}{\sum }}P_{i}\log_{2}P_{i}$$

The smaller the value of *PE_M_*, the more regular and more deterministic is the time series. Contrarily, the closer to 1 *PE_M_* is, the noisier and more random the time series is.

### 2.2. Channel Selection by KNN Based on Genetic Algorithm

Noise and redundant data points in signals can render information on the training of the method irrelevant. For effective and efficient EEG signal analysis, identifying the channels that contribute most to the prediction outcomes is crucial. A genetic algorithm (GA), developed by John Holland et al. in 1970s [35] is also applied in this research. A GA is a search heuristic that imitates the process of Charles Darwin’s theory of natural selection, in areas such as inheritance, mutation, selection, and crossover.

For feature selection, ‘mutation’ in GA means switching features on and off. ‘Crossover’ means interchanging the used features. In this paper, the selection is based on the accuracy of the KNN classification performance. KNN is a supervised learning algorithm, and it is one of the most important non-parameter algorithms in the pattern recognition field [36]. The training samples themselves generate the classification rules without any additional data. The KNN classification algorithm predicts the test sample’s category according to the K training samples, which are the nearest neighbors to the test sample, and judges the category with the most significant probability [36].

**Figure 2:**
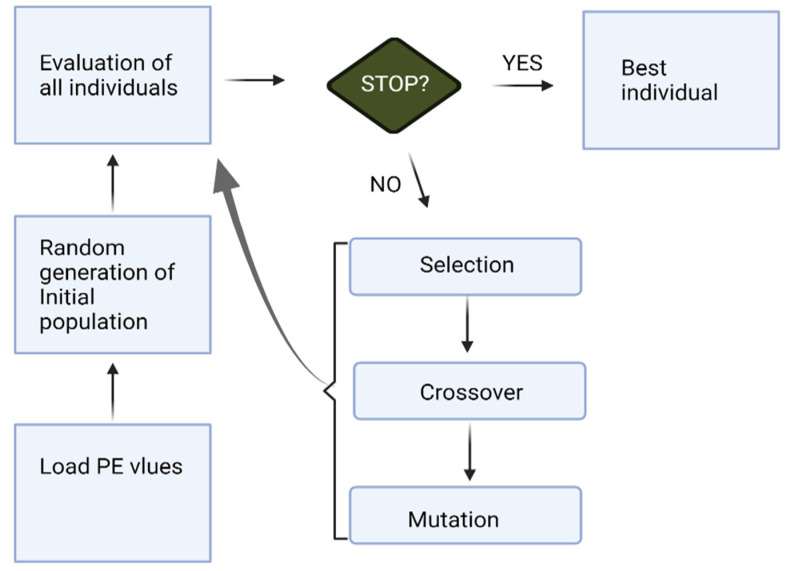
The process of KNN-GA.

The overall process of KNN-GA for a channel selection works as follows in this study (Figure 2):

1. Load the PE values (Section 2.2) of each channel.
2. KNN-GA begins with a set of individual subjects, which are the total population (all individuals). A subject is described by a set of parameters (channels in this research) noted as Genes. Genes are combined into a string to form a Chromosome (any possible solution). The population size is 20, and the minimum number of Genes is one.
3. Then each Chromosome in the population is evaluated by the fitness function (KNN in this paper) to test how well it predicts pre-ictal periods. It gives a fitness score (maximum: infinity) to each subject.
4. Now the selection operator chooses some of the Chromosomes for reproduction based on a probability distribution. We set 0.9 for the initial probability. For example, if *f*(*x*) is a fitness function, then the probability that chromosome *C_X_* is chosen to reproduce is:
  (2) $$p\left(C_{x}\right)=\frac{f\left(C_{x}\right)}{\sum_{i=1}^{N_{pop}}f\left(C_{i}\right)}$$
  where *Npop* is the number of Chromosomes in the population.
5. Next, we mix Chromosomes for crossover (type: uniform, crossover probability: 1.0). Each Gene is selected randomly from one of the corresponding genes of the parent Chromosomes.
6. The final step is to apply random mutations. For each Gene that we are to copy to the new population, we allow a small probability of error (0.01 in this paper).
7. Repeat from step 2 until the population converges (does not produce offspring which are significantly different from the previous generation). It can then be said that the genetic algorithm has provided a set of solutions to our problem (maximum number of generations: 30).

### 2.3. Selected Channels Validation by a SVM Model

**Figure 3:**
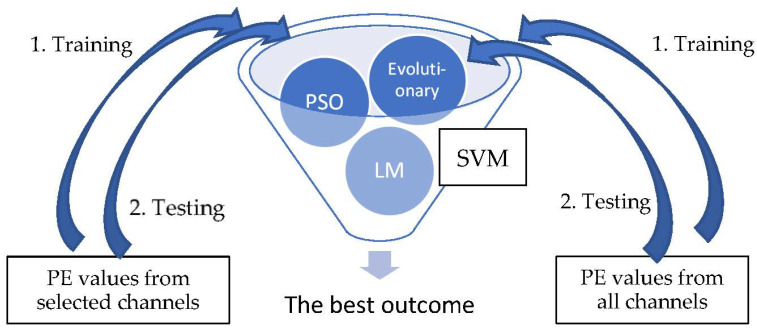
SVM classification.

Following channel selection, a SVM is used to classify the patterns into pre-ictal and normal periods. There are three types of optimization method for the SVM used in this research: Lagrange multiplier (LM), evolutionary and Particle Swarm Optimization (PSO). The PE values of the selected channels by KNN-GA were trained and tested for each of the three types of SVMs, and the best result was selectively adopted. The PE values of all channels were also derived through the same process. The detailed steps are demonstrated below (Figure 3).

## 3. Results 

### 3.1. The Experimental Data and Clinical Consideration

The experimental data came from CHB-MIT Scalp EEG Database [37]. This the data of this database is collected at the Children’s Hospital Boston. It consists of EEG recordings from pediatric subjects with intractable seizures. Subjects were monitored for several days following withdrawal of anti-seizure medication in order to characterize their seizures and assess their candidacy for surgical intervention. Recordings, grouped into 24 cases, were collected from 24 subjects (5 males, ages 3–22; 18 females, ages 1.5–19). Each case (chb01, chb02, etc.) contains 9 to 42 continuous .*edf* files from a single subject. The characteristics of each patient and the patient’s data are summarized below (Table 1).

**Figure 4:**
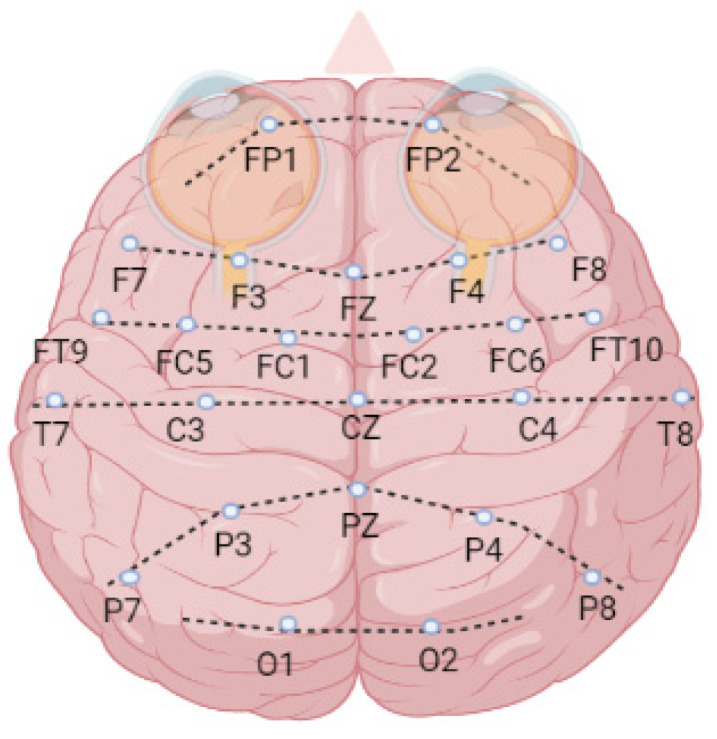
The brain surface map of EEG channels.

The 24 patients’ EEG signals with a 256 Hz sampling rate were recorded using 23 channels which are FP1-F7 (1), F7-T7 (2), T7-P7 (3), P7-O1 (4), FP1-F3 (5), F3-C3 (6), C3-P3 (7), P3-O1 (8), FP2-F4 (9), F4-C4 (10), C4-P4 (11), P4-O2 (12), FP2-F8 (13), F8-T8 (14), T8-P8 (15), P8-O2 (16), FZ-CZ (17), CZ-PZ (18), P7-T7 (19), T7-FT9 (20), FT9-FT10 (21), FT10-T8 (22), and T8-P8 (23). The letter notations are—FP: frontopolar, F: frontal, T: temporal, O: occipital, C: central, and P: parietal (Figure 4).

**Figure 5:**
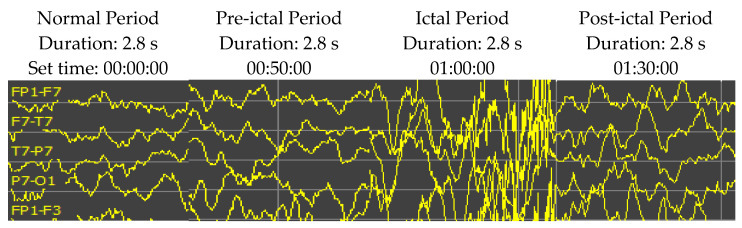
An example of EEG recordings (Patient ID 1, channels of FP1-F7, F7-T7, T7-P7 and P7-O1) over time showing the activity from the EEG signals at the normal, pre-ictal, ictal, and post-ictal periods. The patient was an 11-year-old female. The sampling rate is 256 Hz. The vertical scale is 50 µV.

Epileptic EEG signals are typically classified into four periods: normal, pre-ictal, ictal, and post-ictal periods (as shown in Figure 5). In some experimental results, the high accuracy rate might not be impressive when available normal period data are surplus and the pre-ictal period signals occupy only a tiny fraction of the testing dataset. Thus, this research restricts the ratio of normal to pre-ictal training/testing data up to 10:1. Selecting segments of EEG signal recording for the analysis is one of the significant problems of seizure prediction research. The seizure prediction horizon (SPH) is the period between the seizure alarm sign and the beginning of seizure occurrence. Therefore, the SPH prerequisites are to be designated before assessing the analysis. The size of the SPH has been reported to be between a few minutes and several hours before a seizure onset. The standard size is still a debatable question. This research set an SPH of 10 min (2.8 s duration) for both training and testing.

Each patient dataset contains data points of 17–154 h. Data samples of a normal period (2.8 s duration) are randomly selected in each hour of the 17–154 h duration. In summary, the samples are collected from:

- Pre-ictal period: 10 min before a seizure onset.
- Normal period: between pre-ictal and post-ictal periods (30 min after a seizure onset).

### 3.2. Validation of the Channel Selection Technique

**Figure 6:**
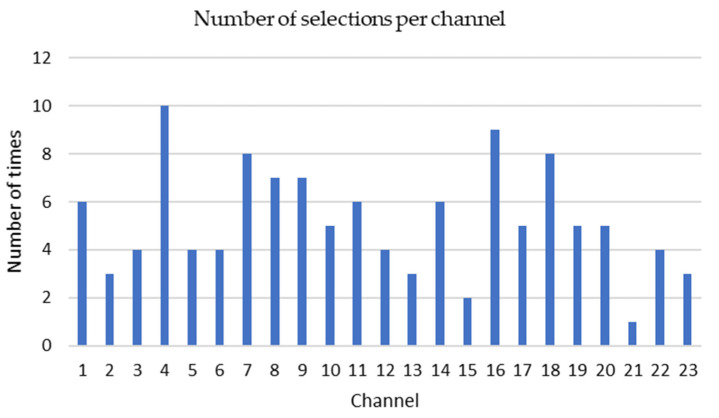
The number of times and each channel from 1 to 23 has been selected. The vertical axis shows how many times one given channel has been selected. Channel 1: FP1-F7, 2: F7-T7, 3: T7-P7, 4: P7-O1, 5: FP1-F3, 6: F3-C3, 7: C3-P3, 8: P3-O1, 9: FP2-F4, 10: F4-C4, 11: C4-P4, 12: P4-O2, 13: FP2-F8, 14: F8-T8, 15: T8-P8, 16: P8-O2, 17: FZ-CZ, 18: CZ-PZ, 19: P7-T7, 20: T7-FT9, 21: FT9-FT10, 22: FT10-T8, 23: T8-P8.

The KNN-GA algorithm selected three to eight channels among 23 channels based on the PE values from each patient’s EEG signals. The most frequently selected channels are P7-O1 (10 times), P8-O2 (9 times), C3-P3 (8 times) and CZ-PZ (8 times) from 22 patient datasets (Figure 6).

The efficiency of a seizure prediction algorithm is determined by the prediction rate, accuracy, sensitivity, and specificity. The prediction rate refers to how many predictions are correctly made out of the total number of ictal occurrences in the testing set. Sensitivity is the percentage of the true pre-ictal prediction, and specificity is the percentage of the true normal period prediction (Table 2). Table 3 presents the performance of the selected channels and all channels based on the SVM classification testing for the 22 patients in the CHB-MIT Scalp EEG Database.

The prediction rate average of the selected channels from 22 patients is 92.42%, while that of all channels from 22 patients is only 71.13%, an improvement of 29.93%. The accuracy average of the selected channels is 74.60%, and that of all channels is 67.46%. The sensitivity and specificity completed by the selected channels testing also show a higher rate (average 69.51% and 73.14%, respectively) than all channels testing (average 56.25% and 69.29%, respectively). On average, the accuracy, sensitivity, and specificity with selected channels are improved by 10.58%, 23.57%, and 5.56%, respectively. The analysis of variance (ANOVA) tests also confirm that the accuracy and sensitivity using the selected channels from the SVM testing result are significantly higher than those using all channel testing results (at *p* < 0.01 and *p* < 0.05, respectively) (Table 4). The standard deviations of the accuracy, sensitivity, and specificity from the selected channels testing for the 22 patients are smaller (15.36, 25.03, and 20.81, respectively) than from all channel testing (Table 4). In addition, the execution time of the SVM model is almost instantaneous (10–500 milliseconds) in many patients’ cases. Nevertheless, the average percentage of computational runtime saved by channel selection is 42%.

**Figure 7:**
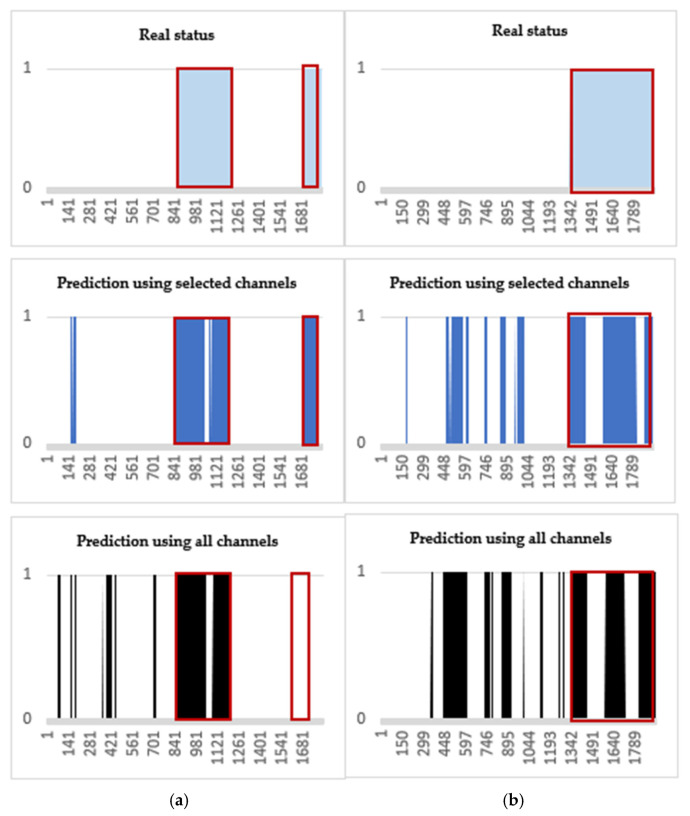
Visual comparisons for the SVM testing results. Blue-colored area with red outlines represents the SPH (10 min), i.e., alarming at 10 min before the seizure onsets. (**a**) Patient ID 20: a total of 4 seizure occurrences in a period of 24 h. (**b**) Patient ID 3: a total of 3 seizure occurrences.

Two-dimensional area graphs are also added to view the numerical results visually (Figure 7). In Figure 7a,b, the blue shapes with red outline (pre-ictal period) of “Real status” are closer to the blue shapes of “Prediction using the selected channels” than the black shapes of “Prediction using all channels”. Thus, the figures demonstrate that using the selected channels can better predict the pre-ictal period than using all channels.

## 4. Discussion

Seizures can occur anywhere in the brain, but for children, they frequently occur in the temporal and frontal lobes, affecting the functions these regions control [38]. Three to eight channels among 23 channels were selected for each subject by KNN_GA based on PE values of epileptic EEG signals. The most frequently selected channel was P7-O1 (10 times), which is located at the scalp of the parietal and occipital lobes of the brain. However, the total number of channels connected to the frontal and temporal lobes region is much higher than that of the parietal and occipital region channels. Consequently, the number of selected frontal and temporal lobes region channels is higher.

The patient-specific channel selection technique improves the prediction rate by 29.93% and the accuracy, sensitivity, and specificity by 10.58%, 23.57%, and 5.56%, respectively. The average accuracy, sensitivity, and specificity of the SVM testing are 74.60%, 69.51%, and 73.14%, respectively, and with all channels, they are 67.46%, 56.25%, and 69.29% in this research into epileptic seizure prediction. In particular, the true pre-ictal prediction rate (sensitivity) of the classification with the selected channels is considerably higher than that with all channels. The corresponding standard deviations are also smaller than those using all channels, demonstrating that tailored channels are more robust in optimizing seizure prediction rates. With the selected channels, the highest accuracy, sensitivity, and specificity rates are 97.28% (patient ID 1), 99.17% (patient ID 7), and 100% (patient ID 1), respectively. On the other hand, patient ID 17 and ID 24 cases achieved poor accuracy (under 50%) despite having high sensitivity.

There are a couple of limitations for the proposed approach. (1) Based on the results from different subjects (such as Patients 17 and 24), it is observed that the patterns of PE values during the nighttime are similar to the patterns of PE values during the pre-ictal period. This phenomenon may affect the predication accuracy. In reality, it is difficult to verify whether a patient is sleeping or just at rest during the nighttime. (2) It is possible that the starting point of the preictal periods are likely not the same for all patients. In this research, the SPH is set to 10 min for all subjects during the model training, while the SPH could be any time period (e.g., several hours).

This research aims to reduce the complexity of feature extraction and classification steps in predicting seizures while a high accuracy is retained and the computation time is significantly reduced. The average execution time by using the selected channels was only 47.09% of that by all channels. For Patient IDs 1, 8, 19, and 20, more than 90% validation accuracy, sensitivity, and specificity rates with just a few selected channels are obtained in this research method. The results demonstrate that the proposed EEG channel selection method with a suitable classification algorithm (SVM in this paper) can increase real-time seizure prediction accuracy.

## 5. Conclusions

In this paper, we recognize that the patterns of epileptic seizure occurrences are patient specific. The key issue is to discern which regions of the brain are most relevant to the seizure onsets for a specific patient. The most frequently selected channel was P7-O1 (10 times). However, many EEG channels were connected to the temporal and frontal lobes, which frequently causes seizures in children.

After finding the suitable channels for each patient through the KNN-GA algorithm, the SVM training and testing based on PE values of epileptic EEG signals exhibit more accurate outcomes of seizure prediction and less computation load than with all 23 channels. Consequently, fewer patient-dependent EEG channels can contribute to essential aspects of seizure prediction analysis, such as less EEG electrodes required on the scalp and more accurate mobile real-time seizure predictions.

## Figures and Tables

**Table 1:** The characteristics of each patient and the patient’s data.

Patient ID	Gender	Age	Number of Seizures	Length of Records (Hours)
1	F	11	7	45.00
2	M	11	3	39.57
3	F	14	7	57.87
4	M	22	4	154.41
5	F	7	5	38.09
6	F	1.5	10	89.25
7	F	14.5	3	67.23
8	M	3.5	5	26.38
9	F	10	4	65.92
10	M	3	7	72.49
11	F	12	3	73.30
12	F	2	40	NA
13	F	3	12	NA
14	F	9	8	41.50
15	M	16	20	62.29
16	F	7	10	17.03
17	F	12	3	34.11
18	F	18	6	62.85
19	F	19	3	61.58
20	F	6	8	41.43
21	F	13	4	55.71
22	F	9	3	75.93
23	F	6	7	70.90
24	NA ^1^	NA	16	NA

^1^ Not available. Not specified.

**Table 2:** Accuracy, sensitivity, and specificity.

	True Pre-Ictal Period	True Normal Period
Predict pre-ictal period	I	II
Predict normal period	III	IV

Accuracy = (I + IV)/(I + II + III + IV). Sensitivity = I/(I + III). Specificity = IV/(II + IV).

**Table 3:** The performance of the selected channels and all channels based on the SVM classification testing for 22 patients.

Patient ID	Recording Duration (Hours)	Number of Seizures		Selected Channels ^1^	Test Results (Selected Channels/All Channels) ^2^				Execution Time s: Second(s) ms: Milli-Second(s)	SVM Optimization Methods
		Train	Test		Prediction Rate (%)	Accuracy (%)	Sensitivity (%)	Specificity (%)		
1	45.00	4	3	*4*, *11*, *16*, *18*	**100**/100	**97.28**/100	**93.66**/100	**100**/100	**32 ms** (30% ^4^)/46 ms	LM
2	39.57	1	2	*5*, *6*, *8*, *11*	**100**/75	**54.27**/30.85	**50.00**/38.02	**56.40**/27.27	**47 ms** (40%)/78 ms	Evolutionary
3	57.87	4	3	*4*, *8*, *14*, *16*, *18*, *19*, *20*, *23*	**100**/67	**77.89**/70.51	**70.91**/62.81	**81.07**/74.00	**121 s** (24%)/159 s	Evolutionary
4	154.41	2	2	*8*, *10*, *14*, *17*, *18*, *19*	**100**/50	**80.79**/76.82	**45.45**/24.79	**84.71**/82.60	**44 s** (15%)/52 s	PSO
5	38.09	2	3	*1*, *7*, *9*, *13*, *16*	**100**/50	**58.19**/55.19	**90.12**/46.88	**38.65**/60.27	**1 s** (80%)/5 s	Evolutionary
6	89.25	6	4	*6*, *9*, *14*, *16*, *18*	**100**/75	**66.04**/58.38	**71.19**/59.27	**57.02**/56.82	**250 ms** (33%)/375 ms	LM
7	67.23	1	2	*4*, *11*, *16*, *18*	**100**/50	**83.61**/80.17	**99.17**/0.00	**80.50**/96.20	**16 ms** (48%)/31 ms	LM
8	26.38	2	3	*8*, *14*, *17*, *19*	**100**/100	**95.73**/65.56	**87.19**/74.38	**100**/62.40	**16 ms** (48%)/31 ms	LM
9	65.92	3	1	*4*, *11*, *16*, *18*, *20*	**100**/100	**69.01**/61.98	**34.71**/68.60	**72.82**/61.25	**31 ms** (83%)/187 ms	LM
10	72.49	3	4	*4*, *8*, *11*, *16*, *18*, *20*	**75**/75	**65.97**/44.03	**80.17**/74.10	**60.64**/61.98	**17 s** (29%)/24 s	PSO
11	73.30	1	1	*3*, *4*, *7*, *8*, *10*, *17*, *21*	**100**/0	**84.55**/71.98	**50.41**/0.00	**88.34**/47.70	**16 ms** (36%)/47 ms	LM
12	NA ^3^	NA	NA	*NA*	NA	NA	NA	NA	NA	NA
13	NA ^3^	NA	NA	*NA*	NA	NA	NA	NA	NA	NA
14	41.50	5	3	*1*, *2*, *3*, *5*, *7*, *9*, *23*	**33.3**/33.3	**61.67**/60.27	**32.51**/18.73	**70.41**/72.73	**64 s** (11%)/72 s	Evolutionary
15	62.29	6	7	*1*, *7*, *17*, *10*, *16*, *22*, *CP2-Ref*	**100**/42.9	**75.48**/63.87	**69.42**/22.41	**80.33**/97.02	**1** s (80%)/5 s	LM
16	17.03	2	3	*1*, *2*, *5*, *7*, *9*, *10*, *15*, *16*	**100**/100	**69.97**/69.83	**100**/100	**54.96**/54.75	**93 ms** (69%)/297 ms	LM
17	34.11	2	1	*1*, *9*, *12*	**100**/100	**45.45**/46.38	**100**/87.60	**37.66**/40.50	**31 ms** (34%)/47 ms	LM
18	62.85	2	4	*4*, *7*, *13*, *20*, *22*	**75**/75	**71.63**/66.39	**42.77**/50.00	**86.05**/74.59	**15 s** (6%)/16 s	PSO
19	61.58	1	1	*4*, *6*, *14*	**100**/100	**94.86**/96.05	**90.08**/100	**95.45**/95.56	**16 ms** (57%)/37 ms	LM
20	41.43	4	4	*3*, *4*, *6*, *7*, *12*, *15*	**100**/75	**94.63**/87.02	**90.63**/88.43	**96.34**/86.42	**20 s** (9%)/22 s	PSO
21	55.71	2	2	*4*, *7*, *13*, *19*, *20*, *22*	**50**/50	**69.35**/49.44	**42.15**/23.14	**75.39**/55.28	**297 ms** (62%)/781 ms	LM
22	75.93	2	1	*1*, *2*, *9*, *12*, *23*	**100**/100	**88.55**/80.05	**24.79**/22.31	**99.17**/89.67	**16 ms** (48%)/31 ms	LM
23	70.90	4	3	*8*, *10*, *14*, *17*, *18*, *19*	**100**/100	**87.40**/100	**96.69**/100	**59.50**/100	**15 ms** (6%)/16 ms	LM
24	NA	10	5	*3*, *5*, *9*, *11*, *12*, *23*	**80**/80	**48.91**/49.44	**67.27**/76.03	**33.61**/27.27	**312 ms** (38%)/500 ms	LM

^1^ Channels, 1: FP1-F7, 2: F7-T7, 3: T7-P7, 4: P7-O1, 5: FP1-F3, 6: F3-C3, 7: C3-P3, 8: P3-O1, 9: FP2-F4, 10: F4-C4, 11: C4-P4, 12: P4-O2, 13: FP2-F8, 14: F8-T8, 15: T8-P8, 16: P8-O2, 17: FZ-CZ, 18: CZ-PZ, 19: P7-T7, 20: T7-FT9, 21: FT9-FT10, 22: FT10-T8, 23: T8-P8. ^2^ Bold represents the testing results of the selected channels. ^3^ Not available. Not possible to match training and testing sets as the channels were frequently changed during the EEG recording—the recordings may be contaminated. ^4^ The percentage of computational runtime saved by channel selection. The average is 42%.

**Table 4:** The ANOVA test results by the SVM classification.

	Accuracy		Sensitivity		Specificity	
	Selected Channels	All Channels	Selected Channels	All Channels	Selected Channels	All Channels
N	22	22	22	22	22	22
∑X	1641.23	1484.21	1529.29	1237.50	1609.02	1524.28
Mean	74.60	67.46	69.51	56.25	73.14	69.29
*σ*	15.36	18.36	25.03	33.44	20.81	22.52
*p*-value	0.002699		0.033532		0.339937	
*F*-ratio	11.5588		5.17403		0.95353	
	significant at *α* = 0.01		significant at *α* = 0.05		not significant at *α* = 0.05	

## Data Availability

The data and materials used in this study are available at the University of Southern Queensland under the research data management policy.
